# Treatment of alcohol dependence in Swedish primary care: perceptions among general practitioners

**DOI:** 10.1080/02813432.2021.1922834

**Published:** 2021-06-21

**Authors:** Karin Hyland, Anders Hammarberg, Sven Andreasson, Maria Jirwe

**Affiliations:** a Centre for Psychiatry Research, Department of Clinical Neuroscience, Karolinska Institutet, Stockholm, Sweden; b Centre for Dependency Disorders, Stockholm Health Care Services, Stockholm County Council, Stockholm, Sweden; c Department of Global Public Health, Karolinska Institutet, Stockholm, Sweden; d Department of Health Sciences, Red Cross University College, Huddinge, Sweden

**Keywords:** Alcohol dependence, primary care, GPs’ perceptions, internet-based Cognitive Behavioral Treatment (iCBT), qualitative study

## Abstract

**Objective:**

To describe general practitioners’ (GPs) attitudes to the management of patients with alcohol dependence in primary care and current treatment routines and their view on a new treatment approach; internet-based Cognitive Behavioral Therapy (iCBT).

**Design:**

A qualitative interview study with ten GPs participating in a randomized controlled trial. The interviews were analyzed using qualitative content analysis.

**Setting:**

The participating GPs were recruited via purposeful sampling from primary care clinics in Stockholm.

**Subjects:**

The GPs were participants in an RCT investigating if iCBT when added to treatment as usual (TAU) was more effective than TAU only when treating alcohol dependence in primary care.

**Results:**

The GPs found alcohol important to discuss in many consultations and perceived most patients open to discuss their alcohol habits. Lack of training and treatment options were expressed as limiting factors when working with alcohol dependence. According to the respondents, routines for treating alcohol dependence were rare.

**Conclusion:**

GPs believed that iCBT might facilitate raising questions about alcohol use and thought iCBT may serve as an attractive treatment option to some patients. The iCBT program did not require GPs to acquire skills in behavioral treatment, which could make implementation more feasible.

**KEY POINTS**
- Alcohol dependence is highly prevalent, has a large treatment gap and is relevant to discuss with patients in many consultations in primary care.
- This study is based on interviews with 10 GPs participating in a randomized controlled trial comparing internet-based Cognitive Behavioral Therapy (iCBT) for alcohol-dependent patients to treatment as usual.
- GPs viewed alcohol habits as important to discuss and they perceived most patients are open to discuss this.
- The access to iCBT seemed to increase GPs’ willingness to ask questions about alcohol and was viewed as an attractive treatment for some patients.
- The iCBT program did not require GPs to acquire skills in behavioral treatment, which might be timesaving and make implementation more feasible.

## Introduction

Alcohol dependence is one of the most prevalent mental disorders and is highly prevalent worldwide, but most individuals with alcohol dependence are not reached with treatment [1]. In Sweden the prevalence of hazardous drinking is estimated to be 20% among men and 13% among women, using the AUDIT-C (Alcohol Use Disorders Identification Test – consumption) instrument. The prevalence of alcohol dependence, based on DSM-IV criteria, was estimated to 4% in a Swedish study [2] and to 5.1%, based on DSM-5 criteria in a WHO study [3].

Excessive alcohol consumption is a significant risk factor for several of the major health problems, for example, cardiovascular diseases, cancers and psychiatric disorders. In a systematic analysis for the Global Burden of Disease Study 2016 alcohol is identified as a major risk factor for the global disease burden and causes substantial health loss [4]. Alcohol dependence has the greatest impact on the burden of disease and on public health among the alcohol use disorder categories [5]. The level of consumption is highly correlated to the number of diagnostic criteria for alcohol dependence fulfilled [6]. More importantly, the higher the level of consumption, the stronger the health effects of a given reduction [7]. Studies on the health-protective effects from alcohol have been published over the years, but a number of recent reports conclude that the risk of all-cause mortality rises with increasing level of consumption, with little or no protection from moderate consumption and that the level of consumption that minimizes health loss is zero [4,8]. A large proportion of individuals with alcohol dependence are already present in primary care for the treatment of other conditions. For many of these, high alcohol consumption has a negative impact on treatment outcomes [1]. According to WHO alcohol consumption is associated with more than 200 health conditions [9].

Alcohol dependence has the widest treatment gap between the number of individuals affected and the number in treatment among mental disorders with only around 10–20% seeking treatment [10,11]. Most people with alcohol dependence have a moderate level of dependence; fulfilling three or four of the seven DSM-IV (Diagnostic and Statistical Manual of Mental Disorders, Fourth Edition) criteria for dependence, and are socially stable [2]. In this group, many individuals are concerned about the consequences of their drinking and want to reduce their consumption [12]. However, alcohol dependence is more stigmatized than most other health problems [13]. The majority of individuals with alcohol dependence are reluctant to seek treatment in specialized care largely due to the stigma related to alcohol problems, but studies indicate that people with alcohol dependence are more positive to seek treatment in primary care [12,14]. Individuals with moderate dependence can be helped with limited treatment within primary care [15].

Alcohol consumption is thus relevant to discuss in many consultations in primary care [16]. GPs are at present reluctant to engage in this area mostly due to time constraints and uncertainty regarding their competence in this field [17–20]. The integration of alcohol treatment into the routines in primary care is limited [21]. Most previous studies in primary care have evaluated screening and brief intervention (SBI) in managing hazardous and harmful alcohol consumption, but SBI has not been successfully implemented [22,23]. Treatment for alcohol dependence in primary care has been studied to a lesser extent and SBI as an intervention has not shown to be efficacious for alcohol-dependent patients [24]. There is furthermore no evidence that SBI increases treatment-seeking in specialized care [25]. However, there are some studies showing successful treatment for alcohol dependence in primary care with acamprosate and naltrexone [26,27]. Nevertheless, only a minority of alcohol-dependent patients are reached with medication [28]. A study where general practitioners (GPs) were given a one-day training, including brief interventions and pharmacological treatment for alcohol dependence, found the treatment given in primary care as effective as treatment given in specialized care [29].

Internet-based interventions are attractive to and reach people with alcohol problems [30]. No studies on internet treatment have focused on alcohol-dependent patients, but a recent meta-analysis showed that internet interventions are effective in curbing different adult problem drinking in both community and health care settings [31]. A qualitative study on patients’ experiences of a computerized self-help program for treating depression in primary care found that internet treatment was attractive to some patients, but not to all [32]. There are no studies on internet treatment for alcohol dependence in primary care. In an ongoing RCT, our research group investigates the effectiveness of an internet-based Cognitive Behavioral Therapy-program (iCBT) for alcohol-dependent patients at primary care clinics in Stockholm [33]. In this RCT we investigated if iCBT facilitated by GPs, when added to treatment as usual (TAU) for alcohol dependence, is more effective than TAU only. GPs’ assessment of the usefulness of this iCBT program is crucial for its future implementation and will be explored in this qualitative study.

## Aim

To describe general practitioners’ (GP) attitudes to the management of patients with alcohol dependence in primary care and current treatment routines and their view on a new treatment approach; internet Cognitive Behavioral Therapy (iCBT).

## Material and methods

The study was a qualitative study based on interviews with GPs. The interviews were analyzed with inductive content analysis [34]. The research team consisted of one psychiatrist working with alcohol treatment (KH), one physician specialized in addiction medicine (SA), one registered psychotherapist specializing in addiction (AH) and one nurse with extensive experience in qualitative research methods (MJ).

### Setting

The participants were GPs from the majority of the primary care clinics in Stockholm County that participated in the ongoing RCT comparing iCBT plus TAU with TAU only. The GPs had experience in the follow-up of patients taking part in the internet study. The participating clinics differed in size and were situated in different socio-economic areas. The routines used for treating alcohol dependence differed both between clinics but also between GPs at the same clinic.

### Procedures

Purposeful sampling was undertaken to ensure information-rich cases [35,36]. This was achieved by recruiting GPs who were active participants in the ongoing RCT, where they had been trained to manage patients with alcohol dependence. The GPs were either contacted by their managers or by the first author (KH) via email. The interviewer was a medical doctor who had met the participants during planning meetings for the RCT.

### Participants

Ten GPs from nine of the 13 primary care clinics that participated in the RCT agreed to participate in the study. Inclusion criteria were to be a GP at a clinic participating in the RCT, with the experience of treating one or more participants in the study. Participating GPs’ age ranged from 41 to 55 years with a median age of 46 years. Their experience as GPs ranged from five to 14 years with a median time of ten years. As many men as women participated.

### Interviews

An open-ended, semi-structured interview guide was used covering current treatment routines and GPs’ attitudes and views on the internet treatment for alcohol dependence. The interview guide was developed by KH and SA and it was piloted with three participants. KH conducted the pilot interviews and a qualitative researcher outside the project gave feedback and some revisions were made to the interview guide. KH conducted all interviews in a place chosen by the participants, usually at their own office. The interviews lasted approximately 30–40 min. After the first two interviews MJ read, and quality approved the interview technique. During the interviews, the questions in the interview guide were followed by supplementary questions to clarify and/or to get a deeper understanding of the subject. Examples of questions asked were: ‘What is your experience in working with alcohol dependence?’; ‘How do you find that patients react when they are asked questions about their alcohol habits?’; ‘What, if anything would make it easier to discuss patients’ alcohol habits?’; ‘Elaborate on your view towards iCBT.’

### Analysis

The interviews were digitally recorded, transcribed verbatim and analyzed with inductive content analysis focusing on the manifest content as described by Graneheim and Lundman [34]. An inductive content analysis was applied where categories are derived from data, that is, the categories are not pre-determined.

The interviews were read several times by KH and MJ as a first step of the analysis. Guided by the aim of the study KH identified meaning units that were condensed and labelled with codes which were then discussed with MJ and altered until agreement was reached. To increase the credibility of the analysis, regular meetings between KH and MJ were held throughout the different stages of the process. Through continuous comparisons of similarities and differences, the codes were abstracted to preliminary sub-categories and categories. The next step was to initiate a reflective interpretative process which involved working back and forth between meaning units, codes, and preliminary sub-categories and categories. In the final step and to further strengthen the credibility of the analysis the sub-categories and categories were reviewed and confirmed by SA and AH (Table 1).

**Table 1.** Example of the analytical process for the three domains with meaning units, codes, sub-categories and categories.

Meaning units	Codes	Sub-categories	Categories
I think I have done it too seldom, and that you, that I sometimes hesitate to ask (about alcohol) because, it is a little difficult or sensitive	Sensitive	Challenges talking about Alcohol	Experiences working with Alcohol Dependence
…ask how much they drink and give suggestions, where you can, for example, suggest to halve the consumption over a period. And I think there are many who accept that.	Goal setting	Patients’ motivation to change their Drinking Habits	Current Routines for Alcohol Treatment in Primary Care
Yes, great advantage, that you just, that you get to choose for yourself when you do it, when you have a moment over and that you can then do it instead of booking another thing that you have to do in some place	Availability	Factors enabling the use of Internet Treatment	Experiences working with Internet Treatment

### Trustworthiness

The analysis was mainly performed by KH, but discussed in daily meetings with MJ during the analysis period to increase the trustworthiness. During these meetings, KH and MJ were reflecting, discussing and revising to refine the analysis by re-labelling and re-sorting codes, sub-categories and categories. AH and SA, with experience from prior qualitative studies, reviewed, considered and confirmed the results. The pilot interviews were not included in the study.

### Ethical considerations

The participants were given written and verbal information about the study and the voluntary nature of their participation. Confidentiality was guaranteed and all participants signed an informed consent to participate. The study was approved by the Regional Ethics Review Board in Stockholm, Dnr 2016/1367-31/2.

## Results

Three main categories emerged from the data: (a) Current routines for alcohol treatment in primary care with the following sub-categories: asking questions about alcohol and patients’ motivation to change their drinking habits; (b) Experiences working with alcohol dependence with the following sub-categories: challenges talking about alcohol, challenges working with alcohol patients and factors affecting patients’ alcohol consumption; and (c) Experiences working with internet treatment with the following sub-categories: factors enabling the use of internet treatment and factors hampering the use of internet treatment (Table 2).

**Table 2.** Overview of categories and sub-categories.

Categories		
Current Routines for Alcohol Treatment in Primary Care	Experiences working with Alcohol Dependence	Experiences working with Internet Treatment
Sub-categories		
Asking Questions about Alcohol Patients’ Motivation to change their Drinking Habits	Challenges talking about Alcohol Challenges working with Alcohol Patients Factors affecting Patients’ Alcohol Consumption	Factors enabling the use of internet treatment Factors hampering the use of Internet Treatment

### Current routines for alcohol treatment in primary care

GPs views on when and how to raise questions about alcohol and how to motivate a patient to change their drinking habits are presented.

#### Asking questions about alcohol

The GPs emphasized that the effects alcohol have on health was important to discuss with their patients. Alcohol was seen as a drug that can both lead to and worsen common somatic and psychiatric conditions and disorders. Therefore, discussing alcohol habits with their patients during their appointments is of great importance according to the GPs.

Alcohol is very important, a natural part of primary care. It is where we should find those (who drink too much), because we work with the diseases that alcohol can cause; long-tern, chronic diseases [8].

The participating GPs usually asked questions about alcohol in the meeting with a new patient and alcohol was considered important among lifestyle habits. The GPs reported that the most common approach was asking questions aimed at quantifying the alcohol consumption. General screening was not applied and patients seeking with acute problems, like a cold, seldom got the question, according to the GPs. Sometimes the GPs forgot to ask or they did not have enough time to ask. The GPs were sometimes unaware that alcohol was the problem, for example, if they did not observe any visible signs of excessive alcoholconsumption. Their own literacy of how alcohol can affect health also had a bearing on whether questions were asked. Some of the GPs believed they do not ask often enough.

If you ask questions about alcohol may depend on how interested and knowledgeable you are…whether you think that alcohol might be important for a certain condition or not [9].

The participating GPs estimated that their frequency of asking questions about alcohol during patient visits varied between 5–50%. They usually only asked when they considered it relevant, meaning when the current problem that brought the patient to the GP could be affected by high alcohol consumption. Another important factor for asking questions about alcohol consumption was whether the GP was able to offer treatment if needed.

We must be able to handle the answers to all questions we ask. And that’s why we don’t ask the question if we don’t know what to do [10].

#### Patients’ motivation to change their drinking habits

Patients’ motivation to receive treatment for alcohol dependence differed according to the GPs. An open conversation about the existing problems due to alcohol was a prerequisite for treatment to take place.

In order to motivate patients to change their alcohol habits, it was common for the GPs to use the current symptoms as a starting point. Asking for permission to inform how alcohol can influence, for example, high blood pressure or depression, was seen as an accessible intervention to create motivation to change.

…you ask the question and some have not reflected so much about it, but then when you address it they start to reflect themselves, so that you are arousing something in them [1].

Other GPs applied a more informative approach and told the patients that it would be a good idea to do something about their alcohol consumption in order to reduce the current somatic or psychiatric symptoms.

We can medicate your blood pressure, but as long as you continue to drink this way, you will counteract it and then we must do something about your drinking as well [2].

Elevated biomarkers indicating heavy drinking and liver pathology could be a way to increase patients’ motivation to change their consumption. It was also seen as a way to follow up treatment effect.

Goal setting was one strategy to encourage patients to cut down their drinking while filling in the alcohol calendar was used to follow up drinking goals.

Sometimes, a small effort is enough…for well-functioning patients who need to be strengthened a little in their own thinking to change their drinking [5].

### Experiences working with alcohol dependence

The participating GPs found alcohol important to discuss, but they viewed alcohol as a challenging and sensitive topic to talk about both for patients and sometimes also for themselves. Insufficient routines and different difficulties working with alcohol habits were described throughout the interviews, for example, on the GP-, patient-, organizational- and educational level.

#### Challenges talking about alcohol

From the GPs perspective, it was common that patients spoke openly about their alcohol habits and their view was that sometimes patients even expected a meticulous doctor to ask questions about alcohol in order to get a broader perspective. The GPs reported they meet emotional expressions such as denial and shame as well as relief, acceptance and openness. Their belief was that patients sometimes denied excessive drinking due to shame and a fear of negatively affecting the doctor–patient relationship. Although both questionnaires and blood tests indicate excessive drinking, the GPs found that it could still be difficult for the patient to speak openly about their alcohol consumption. If the GPs suspected excessive alcohol consumption it could sometimes be easier for the patient to discuss this on another occasion when they were more prepared to talk about it.

Most people are open, and also positive and talk about it… one would get the impression that, when asked the question, it comes with relief for some people [1]

All GPs stated that they were aware of the shame and the guilt linked to excessive alcohol consumption and therefore they were careful not to increase these feelings in their patients. Seeing patients’ alcohol habits affecting the patients’ own lives as well as their loved ones’ lives could arouse frustration among the GPs, but at the same time, GPs belief was that alcohol problems can affect anyone.

I can definitely get extremely frustrated when I see someone completely ruin their life with alcohol in a different way, I think, than if someone has diabetes that they don’t take care of [2].

It was easier to talk about alcohol when this was the primary reason for the visit, which was rarely the case. The GPs also found it easier talking about alcohol with patients taking part in the RCT, where questions about alcohol were raised mandatorily. Some of the GPs also speculated on whether their own perceptions of alcohol and their own alcohol habits could affect the conversation about alcohol.

…one’s own thoughts about alcohol, what one thinks is normal consumption…maybe our prejudices or our own thoughts about this will be reflected in the contact [8].

#### Challenges working with alcohol patients

The clinics seldom had documented routines in the work with alcohol patients. Questionnaires were rarely used and general screening was not applied. The GPs reported that they often were unaware of how their colleagues work with alcohol. The working method seems to depend on one’s own competence and interest regarding the alcohol field.

No, we have no routines…we use ad hoc solutions all the time and we probably also have a little different view on what to do and when to do it [10].

Education and training in how to raise questions about patients’ alcohol habits, how to use alcohol-related diagnoses and to prescribe alcohol medications was a need according to the GPs. The GPs expressed a desire for questions and phrases to use to open the discussion about alcohol.

I would like to practice the conversation technique actually, because I really feel that we fail in the conversation. It tends to be some form of questioning about right or wrong [6].

The GPs reported they seldom use alcohol diagnoses in the records, less frequently compared to the prevalence in the population.

The competence regarding prescribing alcohol medications also varied. Some of the GPs felt competent prescribing acamprosate, naltrexone and disulfiram and were satisfied with their effects. Some of the GPs seldomly prescribed alcohol medications and must refresh their memory before doing so, which made them hesitate to use them. In one of the clinics, there had been no prescriptions at all and in another clinic only the interviewed GP prescribed medications.

About prescribing alcohol medications, I’m a little cautious to do that and I think it’s important that an addiction clinic takes care of that [3].

Another challenge was time constraints and difficulties in offering follow-up visits. One possible solution mentioned was to offer follow-up visits to nurses.

#### Factors affecting patients’ alcohol consumption

All GPs had noticed differences in drinking habits due to gender, economy and what was socially accepted. At the centers located in higher socio-economic areas, the GPs emphasized that patients drank less per occasion, but more regularly and in lower socio-economic areas and groups binge drinking was more common. The GPs reasoned that it was more difficult to identify alcohol problems among patients in higher socio-economic groups, partly due to external signs being less frequent in this group and therefore questions about alcohol were not asked.

…fancy wine drinking among higher social groups is considered cultural, it is not so dangerous if you drink expensive wines [10].

The GPs’ view was that men were more open about their drinking while women seemed to hide their drinking to a greater extent due to stigma. They believed it was more shameful for women to admit they were drinking too much and as a result, it was more difficult to identify alcohol problems in women.

### Experiences working with internet treatment

The iCBT is a self-help CBT program with automated feedback. It includes five modules and can be initiated by the GP or other professions at the clinic. Ideally, follow-up visits for blood samples, additional motivational- and pharmacological treatment can be planned in addition to the iCBT. The GPs pointed out some features that made iCBT useful but also highlighted factors that could complicate the use of iCBT in primary care.

#### Factors enabling the use of internet treatment

Having knowledge about the content of the treatment program and knowing the treatment is evidence-based and effective was considered important by GPs in order to recommend iCBT. GPs believed that the willingness to raise questions about alcohol may increase when iCBT can be offered. This was in line with the previous general statement that it was easier to raise questions about alcohol when having a treatment to offer.

So far, we have not had much to offer except for referral to specialized care, where many patients do not feel they belong [7].

iCBT was also seen to have the potential of attracting patients who would otherwise not participate in treatment due to practical reasons or time constraints. According to the GPs, most patients do not find specialized treatment for alcohol dependence attractive and the GPs found iCBT had the potential to reduce the stigma with its anonymity.

Internet treatment is contemporary and is less stigmatizing for the patient. It is much more accessible, there are less obstacles and practical [4].

The GPs viewed iCBT suitable for patients that are motivated, active and have computer experience. They also thought that iCBT can reduce the workload for the GPs. They considered the treatment has the potential to make patients take responsibility for their own situation, strengthen self-efficacy and support the patient when not in the clinic.

…with the help of this she was able to have a conversation with herself…and that it was very strange when she didn’t drink, what would she do then…and she was worried that she would start eating instead. So, she filled the time with knitting and she said it was so nice that she had to figure out what to do herself [6].

#### Factors hampering the use of internet treatment

GPs feared that resources like time and personnel were needed to make iCBT work as routine, as alcohol treatment is one of many responsibilities in primary care.

Definitely a treatment that could work, but now there would probably not be room for it. We would like to do everything, but we get more and more assignments in primary care, so, not without resources [10].

The GPs stated that a routine that makes the treatment easily available for the GPs, for example, via the data file system, was essential. To receive feedback from the internet platform when treatment ends was also viewed as important as well as resources to organize the treatment. Furthermore, other professions, for example, nurses, should also be engaged in this activity.

…that there is someone at the clinic who may be particularly good at this, that there is a nurse to call if you have questions [6].

The GPs’ perceived that some patients would prefer therapist-assisted internet treatment and others personal contact with the GP. An idea raised to make internet treatment more attractive was adding a chat forum where patients can support each other.

The GPs did not find iCBT to be useful for patients that lack computer experience, for example, some elderly patients and patients with cognitive impairment. However, for patients with multiple diseases in home-care iCBT could be useful.

For the elderly with multiple diseases in home-care most things that are schematic don’t work so well. But on the other hand, there is absolutely nothing for them today and they can’t leave their homes, so this might be good for them [5].

## Discussion

### Statement of principal findings

The aim of this study was to describe GPs’ attitudes regarding working with alcohol dependence, current treatment routines, and their views on iCBT, a new treatment approach to treat alcohol dependence within primary care. The results showed that the GPs considered alcohol dependence to be important to both identify and treat. One way of addressing alcohol that was considered effective by the GPs was to raise the patient’s interest in how alcohol affects their current clinical condition, as previously described by Lid et al. [37]. Alcohol, unlike most other medical conditions, was viewed as a sensitive and stigmatized subject to discuss both for patients and for GPs themselves, which is in line with previous research [12]. Even if this sometimes prevents patients from speaking openly about their alcohol habits the participating GPs found most patients open and positive when questioned about their alcohol consumption, which a recent qualitative study on patients’ experiences also have indicated [38].

Further, the analysis showed that according to the GPs, raising questions about alcohol was facilitated by knowledge about alcohol and its effect on health as well as having been trained in *how* to talk about alcohol. Lastly, it was considered important to have effective treatment options to offer patients and iCBT was a treatment option the GPs found interesting. A need for training in conversation techniques and in the use of pharmacotherapy for alcohol dependence were identified as important by the GPs in order to raise questions about alcohol and offer treatment.

Positive features with iCBT for patients mentioned were; reducing stigma, practical and convenient for active people and strengthening self-efficacy. According to the GPs, resources, like time and personnel, are needed in order to make iCBT useful as a routine. The ability to initiate iCBT from the patient file system and to incorporate automated feedback to the GPs from the treatment platform would make it easier to follow up patients which can further increase the usefulness of iCBT. GPs believed that some patients would prefer personal contact prior to iCBT. Following normal routines in medical care, where all interventions need follow-up visits, the iCBT may also be combined with personal follow-up visits to the GPs or nurses during and after the treatment. The iCBT does not require GPs to learn a new treatment method. As GPs expressed a need for conversational skills, iCBT might hamper their practice and learning. On the other hand, this treatment method would still be available if a GP leaves a clinic and might be time-saving. The availability of iCBT can also result in that treatment as usual is performed to a greater extent, for example, pharmacological treatment and goal-setting. In the RCT, around half of the alcohol-dependent patients were prescribed pharmacological treatment.

General screening was not applied in any participating centers in this study and generally, there was a lack of effective routines concerning alcohol identification and treatment. Time constraints made it difficult to plan revisits. Other professionals, for example, nurses, were seldom involved in this work but were seen by GPs as potential co-workers in identifying and treating alcohol problems.

### Strengths and limitations

A limitation of the study is that only a small group of GPs participated. There is a possibility that the GPs in this study were more positive to iCBT than GPs in general, as they were involved in the RCT and also accepted to take part in this study. However, it is not possible to give a general understanding using a qualitative design but to share experiences, in this case, GPs’ experiences of working with and referring alcohol-dependent patients to iCBT. The participants were selected through purposeful sampling, that is, having experience from the RCT, they were experienced GPs from clinics in different socio-economic areas and also there were as many men as women included in the study to minimize gender bias. The use of quotations enhances the results and facilitates the readers’ ability to judge the credibility and authenticity of the findings. Furthermore, credibility is also enhanced by having one author (KH) conducting the data collection and by having the first (KH) and last author (MJ) closely working together during the entire process of the analysis to further increase the credibility and trustworthiness of the study. Another strength of this study is the exploration of experiences from a novel treatment approach, iCBT, in clinical practice.

### Findings in relation to other studies (differences in results)

Most previous studies in primary care have evaluated SBI that has been found effective in reducing harmful alcohol consumption [23]. However, SBI was not found effective in treating alcohol dependence and did not increase referral to specialized care [24,25]. Likewise, the GPs in this study stated that patients with alcohol dependence seldom are interested in treatment in specialized care. Studies for treating alcohol dependence within primary care are few and have mainly evaluated pharmacological treatment, which is rarely used [26–28]. Patients prefer treatment in primary care to specialized care, especially those with co-occurrent health conditions [38]. This points to the need for GPs to have access to treatment they find applicable and feasible to use. In interviews with GPs taking part in the study for treating alcohol dependence in primary care referred to above [29], the GPs found the stepped-care program as a promising approach that was easy to use [39].

The availability of iCBT facilitated discussions about alcohol among GPs in this study, as they were able to offer a treatment alternative that they found attractive for patients. In order to offer any kind of treatment for alcohol dependence, identification of alcohol habits is the first and crucial step. Without skills in behavioral treatment, there is some reluctance in raising alcohol questions, knowing that a proportion of patients with hazardous use have developed dependence. Thus, iCBT can be a tool both for identifying and a possible treatment option for alcohol dependence in primary care.

### Meaning of the study

Alcohol dependence remains highly stigmatized and the majority of people with alcohol dependence are reluctant to seek treatment. Primary care has for many years been considered the ideal base for treatment, with its broad contact with the whole population. In spite of serious efforts around the world, the integration of alcohol treatment in regular routines in primary care has remained elusive. One reason for this is the perceived lack of expertise among primary care practitioners, leading them to avoid raising the alcohol issue.

What this study adds is the exploration of experiences with a novel treatment approach for alcohol dependence, iCBT.

While acknowledging that the introduction of new technology requires training and resources, having access to an effective internet-based treatment for alcohol dependence was expected to increase the willingness to ask patients about their alcohol habits. This then might constitute a first stepping stone towards a treatment system where primary care is the base of treatment.

## Conclusion

GPs believed that iCBT might facilitate raising questions about alcohol use and thought iCBT may serve as an attractive treatment option to some patients. The iCBT program did not require GPs to acquire skills in behavioral treatment, which could make implementation more feasible.

## References

[1] Rehm J, Allamani A, Elekes Z, et al. Alcohol dependence and treatment utilization in Europe - a representative cross-sectional study in primary care. BMC Fam Pract. 2015;16(1):90. 26219430 10.1186/s12875-015-0308-8 PMC4518612

[2] Andréasson S, Danielsson A-K, Hallgren M. Severity of alcohol dependence in the Swedish adult population: association with consumption and social factors. Alcohol. 2013;47(1):21–25. 23084032 10.1016/j.alcohol.2012.10.001

[3] World Health Organization. Global status report on alcohol and health 2018. Geneva (Switzerland): WHO; 27 September 2018. Available from: https://www.who.int/publications/i/item/9789241565639#:∼:text=Global%20status%20report%20on%20alcohol%20and%20health%202018.,three%20quarters%20of%20these%20deaths%20were%20among%20men

[4] Griswold MG, Fullman N, Hawley C, et al. Alcohol use and burden for 195 countries and territories, 1990–2016: a systematic analysis for the Global Burden of Disease Study 2016. The Lancet. 2018;392(10152):1015–1035. 10.1016/S0140-6736(18)31310-2 PMC6148333 30146330

[5] Rehm J, Gmel GE, Gmel G, et al. The relationship between different dimensions of alcohol use and the burden of disease—an update. Addiction. 2017;112(6):968–1001. 28220587 10.1111/add.13757 PMC5434904

[6] Rehm J, Anderson P, Gual A, et al. The tangible common denominator of substance use disorders: a reply to commentaries to Rehm et al. (2013a). Alcohol Alcohol. 2014;49(1):118–122. 10.1093/alcalc/agt171 24226811

[7] Rehm J, Roerecke M. Reduction of drinking in problem drinkers and all-cause mortality. Alcohol Alcohol. 2013;48(4):509–513. 23531718 10.1093/alcalc/agt021

[8] Wood K, Kaptoge S, Butterworth A, et al. Risk thresholds for alcohol consumption: combined analysis of individual-participant data for 599 912 current drinkers in 83 prospective studies. The Lancet. 2018;391(10129):1513–1523. 10.1016/S0140-6736(18)30134-X PMC5899998 29676281

[9] WHO Library Cataloguing-in-Publication Data Global status report on alcohol and health – 2014 ed.. available from: www.who.int/substance_abuse/publications/global_alcohol_report/msb_gsr_2014_1.pdf?ua=1

[10] Blomqvist J, Cunningham JA, Wallander L, et al. En rapport från “Improving drinking habits”—different patterns of change and the importance of treatment. Social Res Alcohol Drugs. 2007;7:1–147.

[11] Kohn R, Saxena S, Levav I, et al. The treatment gap in mental health care. WHO Bull. 2004;82(11):858–866. PMC2623050 15640922

[12] Wallhed Finn S, Bakshi A-S, Andréasson S. Alcohol consumption, dependence, and treatment barriers: perceptions among nontreatment seekers with alcohol dependence. Subst Use Misuse. 2014;49(6):762–769. 24601784 10.3109/10826084.2014.891616

[13] Schomerus G, Lucht M, Holzinger A, et al. The stigma of alcohol dependence compared with other mental disorders: a review of population studies. Alcohol Alcohol. 2011;46(2):105–112. 21169612 10.1093/alcalc/agq089

[14] Field CA, Klimas J, Barry J, et al. Problem alcohol use among problem drug users in primary care: a qualitative study of what patients think about screening and treatment. BMC Fam Pract. 2013;14(1):98. 23849081 10.1186/1471-2296-14-98 PMC3720559

[15] Storbjörk J, Room R. The two worlds of alcohol problems: who is in treatment and who is not? Addict Res Theory. 2008;16(1):67–84.

[16] Aasland OG, Bruusgaard D, Rutle O. Alcohol problems in general practice. Br J Addict. 1987;82(2):197–201. 3471249 10.1111/j.1360-0443.1987.tb01461.x

[17] Anderson P, Wojnar M, Jakubczyk A, et al. Managing alcohol problems in general practice in europe: results from the European ODHIN survey of general practitioners. Alcohol Alcohol. 2014;49(5):531–539. 25031247 10.1093/alcalc/agu043

[18] Keurhorst M, van Beurden I, Anderson P, et al. GPs’ role security and therapeutic commitment in managing alcohol problems: a randomised controlled trial of a tailored improvement programme. BMC Fam Pract. 2014;15(1):70. 24742032 10.1186/1471-2296-15-70 PMC4021502

[19] Geirsson M, Bendtsen P, Spak F. Attitudes of Swedish general practitioners and nurses to working with lifestyle change, with special reference to alcohol consumption. Alcohol Alcohol. 2005;40(5):388–393. 16043435 10.1093/alcalc/agh185

[20] Nygaard P, Aasland OG. Barriers to implementing screening and brief interventions in general practice: findings from a qualitative study in Norway. Alcohol Alcohol. 2011;46(1):52–60. 21059696 10.1093/alcalc/agq073

[21] Wilson G, Heather B, Kaner N. New developments in brief interventions to treat problem drinking in nonspecialty health care settings. Curr Psychiatry Rep. 2011;13(5):422–429. 21744155 10.1007/s11920-011-0219-x PMC3166704

[22] Van Beurden I, Anderson P, Akkermans RP, et al. Involvement of general practitioners in managing alcohol problems: a randomized controlled trial of a tailored improvement programme. Addiction. 2012;107(9):1601–1611. 22372573 10.1111/j.1360-0443.2012.03868.x

[23] Segura L, Anderson P, Gual A. Optimizing the delivery of interventions for harmful alcohol use in primary healthcare: an update. Curr Opin Psychiatry. 2018;31(4):324–332. 29846264 10.1097/YCO.0000000000000435

[24] Saitz R. Alcohol screening and brief intervention in primary care: absence of evidence for efficacy in people with dependence or very heavy drinking. Drug Alcohol Rev. 2010;29(6):631–640. 20973848 10.1111/j.1465-3362.2010.00217.x PMC2966031

[25] Glass JE, Hamilton AM, Powell BJ, et al. Specialty substance use disorder services following brief alcohol intervention: a meta-analysis of randomized controlled trials. Addiction. 2015;110(9):1404–1415. 25913697 10.1111/add.12950 PMC4753046

[26] Kiritzé-Topor P, Huas D, Rosenzweig C, et al. A pragmatic trial of acamprosate in the treatment of alcohol dependence in primary care. Alcohol Alcohol. 2004;39(6):520–527. 15304381 10.1093/alcalc/agh088

[27] Oslin DW, Lynch KG, Maisto SA, et al. A randomized clinical trial of alcohol care management delivered in department of veterans affairs primary care clinics versus specialty addiction treatment. J Gen Intern Med. 2014;29(1):162–168. 24052453 10.1007/s11606-013-2625-8 PMC3889933

[28] Thompson A, Ashcroft DM, Owens L, et al. Drug therapy for alcohol dependence in primary care in the UK: a clinical practice research Datalink study. PLoS One. 2017;12(3):e0173272. 28319159 10.1371/journal.pone.0173272 PMC5358741

[29] Wallhed Finn S, Hammarberg A, Andreasson S. Treatment for alcohol dependence in primary care compared to outpatient specialist treatment-a randomized controlled trial. Alcohol Alcohol. 2018;53(4):376–385. 29346473 10.1093/alcalc/agx126

[30] Vernon ML. A review of computer-based alcohol problem services designed for the general public. J Subst Abuse Treat. 2010;38(3):203–211. 20015607 10.1016/j.jsat.2009.11.001 PMC2835799

[31] Riper H, Hoogendoorn A, Cuijpers P, et al. Effectiveness and treatment moderators of internet interventions for adult problem drinking: an individual patient data meta-analysis of 19 randomised controlled trials. PLoS Med. 2018;15(12):e1002714. 30562347 10.1371/journal.pmed.1002714 PMC6298657

[32] Holst A, Nejati S, Björkelund C, et al. Patients’ experiences of a computerised self-help program for treating depression – a qualitative study of Internet mediated cognitive behavioural therapy in primary care. Scand J Prim Health Care. 2017;35(1):46–53. 28277055 10.1080/02813432.2017.1288813 PMC5361419

[33] Hyland K, Hammarberg A, Hedman-Lagerlof E, et al. The efficacy of iCBT added to treatment as usual for alcohol-dependent patients in primary care: study protocol for a randomized controlled trial. Trials. 2019;20(1):790. 31888707 10.1186/s13063-019-3902-6 PMC6938008

[34] Graneheim U, Lundman B. Qualitative content analysis in nursing research: concepts, procedures and measures to achieve trustworthiness. Nurse Educ Today. 2004;24(2):105–112. 14769454 10.1016/j.nedt.2003.10.001

[35] Patton MQ. 2002. Qualitative research & evaluation methods. 3rd ed. London (UK): Sage Publications.

[36] Malterud K, Siersma VD, Guassora AD. Sample size in qualitative interview studies: guided by information power. Qual Health Res. 2016;26(13):1753–1760. 26613970 10.1177/1049732315617444

[37] Lid TG, Malterud K. General practitioners’ strategies to identify alcohol problems: a focus group study. Scand J Prim Health Care. 2012;30(2):64–69. 22643149 10.3109/02813432.2012.679229 PMC3378006

[38] O'Donnell A, Hanratty B, Schulte B, et al. Patients’ experiences of alcohol screening and advice in primary care: a qualitative study. BMC Fam Pract. 2020;21(1):68–11. 32321440 10.1186/s12875-020-01142-9 PMC7178930

[39] Wallhed Finn S, Hammarberg A, Andréasson S, et al. Treating alcohol use disorders in primary care – a qualitative evaluation of a new innovation: the 15-method. Scand J Prim Health Care. 2021;39(1):51–59. 33586596 10.1080/02813432.2021.1882079 PMC7971313

